# Psoriasis‐like skin rash triggered by a local infection in a patient with eosinophilic granulomatosis with polyangiitis that was well controlled by mepolizumab treatment

**DOI:** 10.1002/ccr3.7532

**Published:** 2023-06-09

**Authors:** Naho Yokota, Makoto Kondo, Akinobu Hayashi, Masako Ichishi, Yoshiaki Matsushima, Takehisa Nakanishi, Koji Habe, Keiichi Yamanaka

**Affiliations:** ^1^ Department of Dermatology Graduate School of Medicine, Mie University Tsu Japan; ^2^ Department of Pathology Graduate School of Medicine, Mie University Tsu Japan

**Keywords:** EGPA, immunostaining, local infection, mepolizumab, psoriasis vulgaris

## Abstract

**Key Clinical message:**

A patient with eosinophilic granulomatosis with polyangiitis, who was well‐controlled by pharmacotherapy, developed a psoriasis‐like rash due to a local infection. It represents the consequence of an immunologic imbalance.

**Abstract:**

A 48‐year‐old woman was diagnosed with eosinophilic granulomatosis with polyangiitis and treated with mepolizumab. While on treatment, she developed a psoriasis‐like rash on her lower legs following a local ear infection. The rash promptly disappeared after the ear infection cleared and did not recur. The psoriasis‐like rash that appeared was pathologically similar to psoriasis. Excessive production of inflammatory cytokines by the immune system is believed to be involved in the pathogenesis of psoriasis vulgaris. These cytokines are known to induce inflammatory responses and promote epidermal cell proliferation. It is possible that mepolizumab treatment suppressed Th2‐type cytokines, while the local ear infection temporarily induced a strong Th1‐type immunity. This immunologic imbalance may have led to the development of a psoriasis‐like rash.

## Graphical abstract

Histologic features and immunohistochemical findings of large erythema plaques forming on the knees and lower legs.

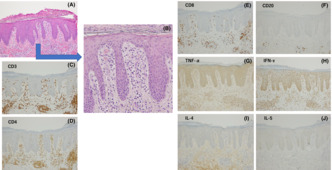

## 1 INTRODUCTION

Eosinophilic granulomatosis with polyangiitis (EGPA) typically occurs in individuals with a history of allergic disease or bronchial asthma and is characterized by systemic necrotizing vasculitis with peripheral blood eosinophilia of unknown etiology. Mepolizumab is a humanized monoclonal antibody against IL‐5, used to treat EGPA and asthma by inhibiting eosinophil proliferation and activity, consequently reducing airway inflammation and EGPA symptoms. Although adverse effects such as headache, hypersensitivity reactions, and injection site reactions are possible, psoriasis‐like dermatitis has not been documented.

We report a case of a patient with well‐controlled EGPA on mepolizumab who developed a psoriasis‐like skin rash on her lower legs due to a local ear infection. This may have been caused by a temporary Th1‐type immune response triggered by the infection, leading to an immunologic imbalance, despite the potential suppression of Th2‐type cytokines by mepolizumab. We suggest that a psoriasis‐like rash can be temporary due to immune balance, as demonstrated in the present case.

## 2 CASE

**FIGURE 1:**
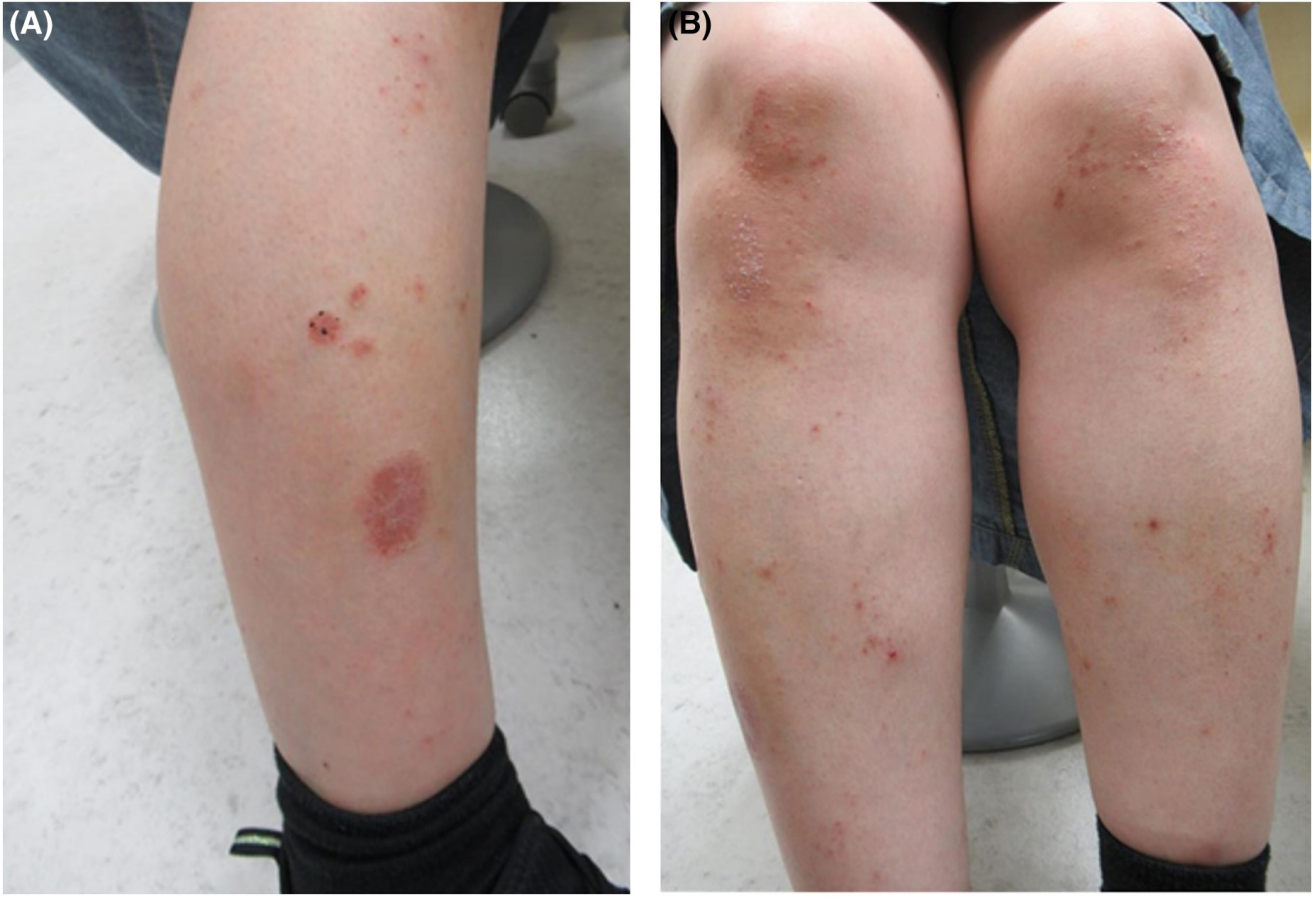
(A, B) Large and small keratotic erythema forming plaque on the knees and lower legs.

**FIGURE 2:**
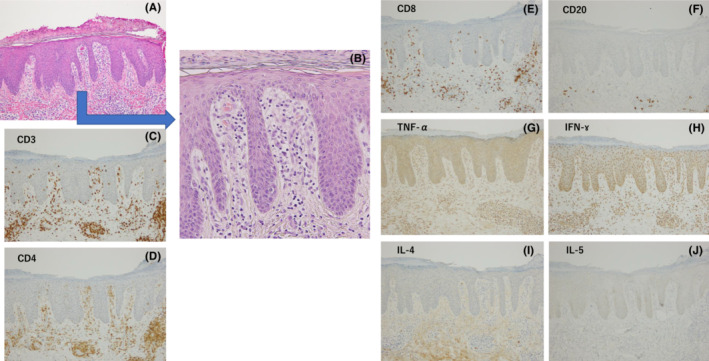
(A, B) Hematoxylin–eosin histological staining of a keratotic erythema specimen obtained from the lower leg (A: magnification: ×100). Histopathologic findings include psoriasis‐like dermatitis with incomplete keratinization, prolongation of epidermal protrusions, telangiectasia of the papillary layer of the dermis, and perivascular infiltration of lymphocytes in the upper dermis. Lymphocytic infiltration is seen in the shallow dermis, extending into the epidermis. Epidermal protrusions showed regular extension. The epidermal processes are regularly prolonged (B: magnification: ×200). (C–E) Immunohistochemical staining for CD3, CD4, and CD8 showing that the markers are highly expressed in the upper dermis (magnification: ×100). (F) Immunohistochemical staining showing very low expression of CD20, a surface marker of B cells (magnification: ×100). (G, H) The inflammatory cytokines IFN‐γ and TNF‐α are highly expressed in the upper dermis layer to the epidermis (magnification: ×100). (I, J) Immunohistochemical staining showing the absence of IL‐4 and IL‐5 (magnification: ×100).

A 48‐year‐old woman developed EGPA with severe numbness in her hands and feet 20 years ago. The patient had bronchial asthma, eosinophilic otitis media, and eosinophilic sinusitis with high IgE levels. She also tested positive for myeloperoxidase‐antineutrophil cytoplasmic antibody (MPO‐ANCA) and proteinase 3 (PR3)‐ANCA at the time of diagnosis of EGPA. Leukocyte and platelet counts were within normal limits, and chest radiographic findings were normal. The patient had been treated with prednisolone (maximum dose of 60 mg/day) for EGPA, but her symptoms remained uncontrolled. Cyclosporine or omalizumab was administered as an additional treatment. The numbness and EGPA symptoms resolved, but the ear and nasal discharge associated with eosinophilic otitis media and eosinophilic sinusitis showed little improvement. When the treatment was switched from omalizumab to mepolizumab, the serum IgE levels decreased, and the symptoms were stably managed, with occasional ear discharge for 6 years. At some point, the patient experienced a local ear infection that resulted in discharge, and a pruritic rash suddenly appeared on her extremities (Figure 1A,B). The patient consulted our department. The patient has no family history of psoriasis vulgaris and has never had similar skin rashes. The patient also had no predisposition to atopic dermatitis or any signs of sebum deficiency. Skin biopsy of keratotic erythema on her lower leg revealed multifactorial epidermal hyperproliferation, regular epidermal process extension, abnormal differentiation of epidermal keratinocytes, and infiltration of inflammatory cells, mainly lymphocytes, in the dermis. These findings were consistent with those of psoriasis vulgaris (Figure 2A,B).

The patient was treated with a strong‐class topical ointment, and the rash disappeared within 1 month. Mepolizumab for EGPA was continued during the period of treatment for the skin rash. To investigate the immunopathologic appearance of temporary keratotic erythema on her extremities, the skin specimens were stained with CD3, CD4, CD8, CD20, TNF‐α, INF‐γ, IL‐4, and IL‐5. Staining results revealed that TNF‐α and IFN‐γ were expressed in the epidermis and upper dermis, whereas IL‐4 and IL‐5 were not detected. CD20‐positive cells were few, while CD3‐, CD4‐, and CD8‐positive cells were abundant (Figure 2C–I). T cells and inflammatory cells were significantly present in the upper dermis, and inflammatory cytokines were abundant in the epidermis. Pathological and immune staining revealed that the same immune response found in most cases of psoriasis vulgaris was also present in the keratotic skin rash. Subsequently, the patient, who continued treatment with mepolizumab, experienced occasional mild ear discharge without infection, but rash‐like psoriasis did not recur.

## 3 DISCUSSION

In this case, a 48‐year‐old woman with well‐controlled EGPA during mepolizumab treatment presented with a psoriasis‐like skin rash triggered by pus discharge from her ear. The appearance of the psoriasis‐like rash in the patient was temporary because it was caused by a local infection and was not an exacerbation of EGPA, nor was it a weakening the effect of mepolizumab. EGPA is a condition characterized by vasculitis, resulting in increased peripheral blood eosinophilia and high IgE levels. EGPA involves an accumulation of immune cells such as eosinophils, T cells, B cells, and macrophages. Inflammation begins when immune cells such as T cells and macrophages enter the lumen of blood vessels and attack vascular endothelial cells and surrounding tissue cells. However, whether these immune responses are entirely responsible for the development of EGPA has not yet been established. Although omalizumab is an anti‐IgE antibody, inhibition of histamine release from mast cells was insufficient to suppress the symptoms of EGPA in the current case. The patient's EGPA symptoms were controlled by the administration of mepolizumab, an anti‐IL‐5 monoclonal antibody. IL‐5 is a key cytokine that plays an essential role in the differentiation, maturation, mobilization, and activation of eosinophils at the sites of allergic inflammation. The immunologic balance in this patient may be due to the suppression of Th2‐type cytokines by the administration of mepolizumab. The immunologic pathogenesis of psoriasis involves the continuous action of TNF‐α, IL‐23, and IL‐17A from upstream to form the skin rash. The psoriasis‐like skin rash was likely caused by the production of inflammatory cytokines by local cytokines from the ear discharge, which strongly stimulated the Type 1 immune system. This phenomenon is supported by the fact that the skin rash disappeared after the ear discharge subsided, and the immunostaining results revealed inflammatory cytokines and inflammatory T cells in the skin specimen from keratotic erythema. Hence, it was assumed that the patient's Th1‐stimulated immune system had calmed down and returned to the Th2 response that had been tipped by the administration of mepolizumab. A psoriasis‐like skin rash has been reported after the administration of anti‐cytokine monoclonal antibodies.^1^, ^2^, ^3^, ^4^ Thus, additional stimuli may cause an immunologic imbalance that results in the appearance of a psoriasiform rash.

In this case, we did not intend to report the failure of EGPA treatment or the development of infection during the treatment of EGPA, but rather the possibility of a psoriasiform skin rash due to such an immune imbalance phenomenon. The sudden appearance of a psoriasiform rash in patients with well‐controlled EGPA through drug treatments should not warrant an immediate change in EGPA treatment, especially assuming that aggravating factors such as local infections, rather than a flare‐up of EGPA symptoms or a weakening of drug effects, are present.

## AUTHOR CONTRIBUTIONS

**Naho Yokota:** Writing – original draft. **Makoto Kondo:** Conceptualization; writing – review and editing. **Akinobu Hayashi:** Writing – review and editing. **Masako Ichishi:** Writing – review and editing. **Yoshiaki Matsushima:** Writing – review and editing. **Takehisa Nakanishi:** Writing – review and editing. **Koji Habe:** Writing – review and editing. **Keiichi Yamanaka:** Writing – review and editing.

## FUNDING INFORMATION

The authors did not receive any financial support for this study.

## CONFLICT OF INTEREST STATEMENT

The authors have no competing interests to declare.

## ETHICS STATEMENT

This study is a medical case report, and ethical approval was not required for this study in accordance with local and national guidelines.

## CONSENT

Written informed consent was obtained from the patient to publish this report in accordance with the journal's patient consent policy.

## Data Availability

All data that support the findings of this study are included in this article.
